# Advanced practice nurses’ and general practitioners’ first experiences with introducing the advanced practice nurse role to Swiss primary care: a qualitative study

**DOI:** 10.1186/s12875-019-1055-z

**Published:** 2019-11-27

**Authors:** Stefan Gysin, Beat Sottas, Muriel Odermatt, Stefan Essig

**Affiliations:** 1 Institute of Primary & Community Care Lucerne, Schwanenplatz 7, 6004 Luzern, Switzerland; 2 Department of Health Sciences and Health Policy, University of Lucerne, Frohburgstrasse 3, 6002 Luzern, Switzerland; 3 sottas formative works, Rue des Epouses 2, 1700 Fribourg, Switzerland

**Keywords:** Advanced practice nurse, Advanced nursing practices, Nurse practitioner, General practitioner, Interprofessional collaboration, Primary care, Family medicine, Switzerland, Qualitative research

## Abstract

**Background:**

Primary care is facing a multimorbid, ageing population and a lack of general practitioners (GPs), especially in rural areas. In many countries, advanced practice nurses (APNs) may be a potential solution for these challenges. Switzerland, however, is in the early stages of APN role development with a handful of pilot projects that are unresearched. Our aim was to explore the experiences of APNs and GPs involved in introducing the APN role to Swiss primary care.

**Methods:**

We organised two focus group discussions with APNs (*n* = 9) engaged in primary care across German-speaking Switzerland and individual interviews with APNs (*n* = 2) and GPs (*n* = 4) from two pilot projects in remote areas. Data analysis followed an exploratory hybrid approach of thematic analysis and was guided by the PEPPA Plus framework.

**Results:**

The analysis resulted in five main themes: The participants considered themselves pioneers developing a new model in primary care, seeking to shape and improve future health care ((1) pioneering spirit). Both nurses and doctors agreed on the additional value of the APN role, a role seen as having more time for and a different approach to patient care, bringing higher quality of care and flexibility to the practice ((2) added value from the APN role). Participants also emphasized the importance of asking for advice when unsure about diagnostic steps or appropriate treatment ((3) awareness of limited knowledge and skills). The main barriers identified included the impression that Swiss doctors have little knowledge about nurses in advanced roles ((4) GP’s lack of knowledge regarding the APN role), and that further regulations will be important to foster role clarity and accountability ((5) political and legal obstacles in introducing the APN role).

**Conclusions:**

The early phase of introducing APNs to Swiss primary care is characterised by heterogeneous, small-scale projects of pioneering GPs and APNs recognising the added value and limits of APNs despite a lack of governance and knowledge regarding the APN role among GPs. Experiences gained from ongoing projects provide elements of good practice for political discussions and regulations.

## Background

Primary care systems worldwide are being challenged by an ageing population with an increasing number of chronic diseases and a higher demand for health care services [1, 2]. At the same time, general practitioners (GPs) are scarce, especially in rural areas [3, 4]. To counteract these challenges, primary care is being reinvented, and many countries have introduced and developed the role of advanced practice nurses (APNs) [5]. Starting in the United States, the role has spread globally and has also been implemented in European countries such as the UK, the Netherlands, and Nordic countries [2, 6]. The International Council of Nurses (ICN) defines an APN as “a registered nurse who has acquired the expert knowledge base, complex decision-making skills and clinical competencies for expanded practice, the characteristics of which are shaped by the context and/or country in which s/he is credentialed to practice. A master’s degree is recommended for entry level” [7]. The most common roles for APNs are the clinical nurse specialist (CNS) with in-depth expertise in a specialised area of practice (e.g. oncology), and the nurse practitioner (NP), with an expanded scope of practice in diagnosing, prescribing, treating, and referring patients [8].

In countries where APNs are well established, they often work as substitutes for doctors in primary care [9]. In a recent review, Laurant et al. [10] assessed their impact on patient outcomes, utilisation and processes of care. Analysing 18 randomised trials, they found that nurses, compared to doctors, have longer consultations, achieve similar or better health outcomes, higher patient satisfaction, and slightly better quality of life for their patients. However, the evidence level for these findings was low to moderate. The effect of nurse-led care on costs remains uncertain due to insufficient evidence. In another review, Jakimowicz et al. [11] found that most GPs believe that APNs have sufficient training to fulfil a role in family practices and GPs are willing to handover individual tasks. Yet, many GPs do not think that nurses are completely autonomous and accountable and therefore able to take full responsibility for patients. The study also revealed that even experienced GPs feel that the role and scope of practice of APNs is still ambiguous.

Switzerland is still at an early stage of APN role development, though the first steps were taken about 20 years ago [12]. The dominating movement has focused on CNS, who work in research or provide leadership at university hospitals. The minor movement has focused on expanding the APN role in primary care; it initially met strong resistance from GPs, but has gained in attention and importance in recent years [13]. It is still rare to find nurses in advanced roles in primary care, but their presence has gradually increased in small-scale projects [2, 14]. To our knowledge, there are presently a handful of pilot projects with APNs in family practices. The current tariff system may hinder APNs from working in primary care. The nationwide uniform fee-for-service system in ambulatory care (TARMED; “tariff medical”) can exclusively be used by medical doctors with a practice authorization [15]. The ambulatory nursing tariff is limited to home care services and not generally applicable to primary care [16]. As a pragmatic solution, most APNs currently use TARMED under the global location number (GLN) of their supervising GPs to reimburse their services at a lower rate than the doctors [unpublished observation]. A lack of accepted educational standards and regulation for APNs may also hinder the extent of their role in practice. While there is consensus among professional organisations that a master’s degree is required, there is no standardized APN curriculum in Switzerland [12]. There is also no legal definition of the APN role or scope of practice, despite national efforts from the nursing side to set up a regulatory framework [8, 17–19]. According to Kieser [20], for the time being the accountability lies with the GP, who delegates the tasks to the APN. Political demands for more regulations were discussed but rejected by the national council in 2016 [21]. An ongoing popular initiative to strengthen the nursing profession is still a subject of debate [22].

Studies about APNs in Switzerland are sparse, particularly regarding their role in primary care [2]. Kambli et al. [23] estimated that 53% of patient consultations in a Swiss urban walk-in clinic could *potentially* be taken over by APNs and concluded that the APN role would contribute meaningfully to the Swiss health care system. Another study by Steinbrüchel et al. [24] examined the views of GPs on *potential* models of collaboration between doctors and nurses. Bryant-Lukosius et al. [8] developed a theoretical framework (PEPPA Plus) for evaluating the impact of different APN roles in various health care settings with the goal of supporting the development of APNs in Switzerland. Yet, it is still unclear how APNs and GPs directly involved in pilot projects experience the introduction of advanced practice nurses in practice. Our aim was to explore their views on introducing the APN role to Swiss primary care.

## Methods

This study followed an exploratory qualitative design with focus groups and individual interviews using a hybrid approach of thematic analysis.

### Setting

Data collection comprised focus group discussions and interviews with APNs and GPs from across German-speaking Switzerland. The focus group discussions involved nurses from various primary care settings, while the individual interviews with APNs and GPs were conducted in two family practices in remote areas of central and north-eastern Switzerland. The project in central Switzerland (“Practice A”) was initiated by the cantonal health department and took place in a small traditional family practice with two GPs nearing retirement. The other project (“Practice B”) was initiated by the owners (two GPs) of an interprofessional group practice. These two projects were selected because they are part of larger, ongoing evaluations. A comparison of the two projects is depicted in Table 1.

**Table 1:** Characteristics of the evaluated projects

	Practice A	Practice B
Location		
Municipality typology^1^	Municipality in a small or outside an agglomeration	Rural, centrally located municipality
Degree of urbanization^2^	Intermediate density area (suburb)	Thinly populated area (rural)
Team		
GP (TEP)	2 (200%)	8 (500%)
APN (TEP)	1 (50%)	1 (50%)
MPA/K (TEP)	5 (380%)	8 (720%)
Other health professionals (TEP)	0 (0%)	3 (170%)
Project		
Launch	August 2017	April 2016
Initiator	Cantonal health department	Practice owners (2 GPs)

*GP* General Practitioner, *APN* Advanced Practice Nurse, *MPA/K* Medical Practice Assistant / Coordinator, Other health professionals = psychologists, physiotherapists, dietitians etc., TEP = Total Employment Percentage

^a^ Gemeindetypologie 2012: www.atlas.bfs.admin.ch/maps/13/de/12359_12482_3191_227/20387.html

^b^ Urbanisierungsgrad 2011 (DEGURBA - Eurostat): www.atlas.bfs.admin.ch/maps/13/de/12476_10444_3191_227/20585.html

### Research team

All authors had profound knowledge of the topic APN in Swiss primary care due to their research activities in this field and knew the participants from the two projects in which they conducted the evaluations. The other study participants were not known beforehand.

### Participants

Purposive sampling was used as a recruitment strategy. Participants were contacted either within the ongoing evaluations (interviews) or via e-mail (focus groups). We received these e-mail addresses by attending conferences, checking practice websites and reaching out to people from universities, universities of applied sciences as well as other projects known to us. At the time of the study, nurses were included if they worked as APNs in primary care, if they were about to start work as APNs, or if they had previous experience in primary care (e.g. during their studies). Other factors such as the length of work experience or gender were not considered in the recruitment process. In the APN role, the participants provided direct patient care and clinical tasks such as physical examination during in-office consultations, and conducted preventive and follow-up home visits to mainly multimorbid elderlies. GPs were eligible if they supervised APNs in their practice. One APN and her supervising GP refused to participate as they were occupied with their own internal evaluation. Nonetheless, we reached a comprehensive sample with APNs from all pilot projects in Swiss primary care. Altogether, nine nurses and four GPs with different backgrounds and experiences participated in this study and no one dropped out. Nine individual interviews and two focus group discussions were conducted. Our research team considered data saturation as achieved within this pioneer setting with only a handful of projects limiting the potential sample size. Characteristics of the participants are shown in Table 2.

**Table 2:** Characteristics of individual participants

	APN (*n* = 9)	GP (*n* = 4)
Age		
Mean (SD)	39.1 (12.4)	57.8 (4.3)
Range	29–59	54–62
Gender		
Female	9 (100%)	0 (0%)
Male	0 (0%)	4 (100%)
Educational level		
Student, MScN	2	.
MScN completed	7	.
In further training	2	.
Further training completed	3	.
Status in primary care		
Currently working in primary care	5	4
Planned projects / internship	4	.
Experience in primary care		
> 20 years	.	2
10–20 years	.	2
1–3 years	4	.
< 1 year	5	.
Type of interview		
Individual interview	2	4
Focus group I	5	.
Focus group II	4	.

*APN* Advanced Practice Nurse, *GP* General Practitioner, *SD* Standard Deviation, *MScN* Master of Science in Nursing, Further training = Diploma of Advanced Studies (DAS); corresponds to 30 ECTS (European Credit Transfer System) with a focus on clinical skills & competencies

### Data collection

Semi-structured face-to-face interviews were conducted with the APN and the two GPs of practice A at the start of the project in August 2017 and repeated 6 months later. Interviews with the APN and the two GPs at practice B were conducted in March and April 2018, respectively, approximately 2 years after the start of the collaboration. Each interview lasted between 30 and 60 min and was carried out at the practice. All interviews were audio-taped and transcribed by the authors (SG, BS, MO). Interview guides were developed to cover aspects considered relevant by international reviews [9, 25] with the goal to cover all important issues (i.e. organisation, collaboration, mentorship, tasks, competencies, acceptance, benefits and reimbursement) without restricting the conversation, and were adapted to the different settings and stages of the projects (see Additional file 1).

The first focus group consisted of five nurses (including the APN from practice B), two moderators (SG, BS) and a minute keeper (MO). The second group consisted of four nurses (including the APN from practice A), one moderator (SG) and a minute keeper (MO). The focus group discussions took place in a neutral setting in April and May 2018, respectively. Both discussions lasted about 2 h and were audio-taped and transcribed by members of the study team (SG, MO). The guides for the discussions contained questions similar to those from the individual interviews but were further developed based on experience from the individual interviews and adjusted to the varying work settings of the participants (Additional file 1).

### Data analysis and rigour

The hybrid approach of thematic analysis by Fereday et al. [26] was chosen to ensure clarity of the data analysis process, trustworthy and scientific rigor. In this method, the deductive a priori template of codes approach of Crabtree and Miller [27] is combined with the data-driven inductive coding of Boyatzis [28], allowing for a balanced identification of emerging themes. Following Fereday’s strict steps, the transcripts were first read and reread to become familiar with the data. Subsequently, a code manual (Additional file 2) was developed with deductive codes based on the PEPPA Plus framework, which includes different perspectives (patients, providers, policy-makers etc.) and evaluation objectives regarding structures, processes and outcomes in APN role development [8]. We chose this guiding framework to explore how theoretical concepts are experienced in practice. The reliability of these codes was tested in two interviews; no modifications were needed. Then, the data were summarized, and initial themes were identified. In a next step, the codes from the manual were applied and additional, inductive codes were assigned. The codes were then connected to identify main themes. Lastly, these themes were corroborated and legitimated. Even though the steps are illustrated separately here, the whole process was iterative and intermediate results of the first author (SG), who performed the coding, were discussed with another author (SE). Disagreements were resolved through consensus. These analyses was supported by MAXQDA 2018 (VERBI Software GmbH, Berlin, Germany).

## Results

The analyses of the interviews and focus group discussions illustrate how theoretical concepts of the PEPPA Plus framework are experienced in practice and resulted in five main themes: (1) “Pioneering spirit”, (2) “Added value from the APN role”, (3) “Awareness of limited knowledge and skills”, (4) “GP’s lack of knowledge regarding the APN role” and (5) “Political and legal obstacles in introducing the APN role”. The quotes that follow are used to exemplify the different themes.

### Pioneering spirit

All nurses felt like pioneers with the opportunity to help develop and shape the role of APNs in Swiss primary care. They emphasized that it is a trial-and-error situation, which requires a certain pioneering spirit in order to help and guide the future generation of nurses. Several APNs also underlined the importance of the role for the future development of the Swiss health care system.

*“This pioneer spirit that we all have at this table is needed.”* (APN, MScN student)

*“I think it’s great that I can participate in this project and help shape the future role of the APN in primary care… because I think it’s really a good thing. I think it’s also important for the whole health care system, for the health care here in this region.”* (APN, in further training)

One APN drew comparisons to the early stages of APN role development in the Anglo-Saxon countries and pointed out that these processes need time. According to her, it might be best to just start and try out this new model in small-scale projects to prove its value in daily practice:

*“It was no different in the Anglo-Saxon world. They also needed their time. My boss told me: Let's just start now. […] This way we can achieve the most and show what we can do and achieve in everyday life. For the patients and also the next generation.”* (APN, further training completed)

Other important aspects that were highlighted by the APNs included the responsibility and pressure that result from this pioneering role. They felt that their new role in primary care draws attention, not only within their own profession, but also in health policy. Therefore, careless behaviour or errors could potentially have a negative influence on the whole movement and hamper its further implementation. However, they also said that these thoughts do not influence their daily work, because there they focus on the patient.

GPs stated that it is interesting to be a pioneer and to help develop a new model in primary care that might help to overcome the current workload and lead to better task sharing among different health care professionals. They admitted that initial extra effort is necessary and that many of their colleagues might not be willing to make that effort or fear their colleagues’ adverse opinion. The doctors confirmed that it is a matter of trial-and-error, and that this was new territory for them, too.

*“To develop something like this has interested me at the end of my professional career… [To] try something that not everyone has done.”* (GP from practice A)

*“Yes, we are doing it now, we are flexible, we are experimenting and yes… maybe we’ll fail but maybe it will work out and prove to be something promising.”* (GP from practice B)

The GPs also stressed that handing over tasks is not easy because family doctors traditionally see themselves as all-rounders and lone fighters. Even the GPs of practice B, who were accustomed to working interprofessionally before the start of the project, admitted that it is sometimes difficult to hand over tasks:

*“I noticed that handing over (tasks) is not easy for us… to get rid of this ‘no one else can do it, that’s why I’m here’ attitude...”* (GP from practice B)

Interestingly, the GPs interviewed did not report feeling much attention or pressure due to their role as pioneers.

### Added value from the APN role

The APNs reported that they have more time for their patients and closer contact to them than the GPs. As another benefit, they mentioned their focus on patients and their daily life, rather than just a condition. The nurses felt that they help provide better care, especially at home, by adding a different approach, e.g. in the areas of lifestyle adaptation and drug compliance. Furthermore, some APNs saw additional value from their role due to their competence in performing tasks such as advance care planning (ACP), technical patient care, or coordination with the social sector, tasks that are often neglected by GPs because of time constraints or lack of knowledge. The nurses also expressed that patients may have fewer inhibitions about addressing certain things in their presence than during consultation with a physician.

*“Asking more detailed questions, taking a closer look. Asking yourself what the disease does to the patient, the family and the environment… plus the connection to some technical tasks, such as taking care of patients with a stoma.”* (APN, in further training)

*“The patients tell us different things than they tell the doctors. Sometimes the patients have too much respect for the doctors and do not tell them certain things… Then the time factor; we have more time. These aspects are an added value, especially when you are at a patient’s home and you see the whole environment… then you might see why a patient is not adherent.”* (APN, MScN)

For the GPs of practice B, the biggest advantages of having an APN included the reduction of their own workload and the additional capacity of the whole practice. They confirmed the added value of home visits performed by the APN and they also felt that patients might be less hesitant to ask “stupid questions” in front of a nurse. Furthermore, they saw an increased quality in their practice due to the APN offering ACP and having more time for the patients.

*“In my 18 years as a GP, I was only once on a home visit to explicitly talk about fall prevention. I know this is something important, but I simply do not have the capacity. […] And apparently, we (physicians) do not find the level of our patients from time to time… They don’t dare to ask us so-called stupid, trivial questions, but they dare to ask the APN.”* (GP from practice B)

*“I was always reluctant to even ask about it (ACP), because I knew I could not manage it in 15 minutes. It is a very important area, but I do not find time for it. There I think she [the APN] is a huge enrichment… I have to say this really is a difference in quality, something that we did not offer before.”* (GP from practice B)

All GPs emphasized the added value of APNs due to their nursing perspective and their way of approaching the patients, e.g. by involving the families and relatives more. The doctors also appreciated the quick availability and flexibility of the APN. Yet, the GPs of practice A said that they see the biggest benefit of the role for a slightly larger practice than theirs:

*“In that respect, I could imagine it in a larger practice. They have more doctors and therefore more patients that the APN can take care of. We are too small…”* (GP from practice A)

### Awareness of limited knowledge and skills

The nurses agreed that it is important to know oneself and one’s own limits in order to avoid harm to the patients. One of the APNs stressed the importance of the capacity for self-reflection, and all nurses valued having a team, and especially an experienced GP in the background.

*“You have to know yourself very well and you have to know your limits. Each one of us has a different focus. You really have to be honest about that. When I'm not sure... You have a team for that, there is a doctor in the background...”* (APN, MScN)

*“I know that I’m not a doctor, I’m a nurse and there are obviously limits. That’s no problem for me, I can ask any time. Actually, there is always a doctor. […] Your self-reflection must be such that you recognise, even under stress, where my limits are.”* (APN, further training completed)

Some APNs also expressed that GPs need to be able to trust them. They need to know that the nurses are aware of their limits and that they do not hesitate to ask for help if necessary. At the same time, it was considered important not to ask about every little detail.

*“The GPs also have to have a certain trust… I work with several doctors and they need to have that trust, that I’m asking when things get tricky. I think it’s good for them to see that I’m actually asking when I’m unsure… But when I ask them about every little thing, I drive them crazy.”* (APN, further training completed)

The GPs confirmed that it is crucial for the APNs to be aware of their limits and to ask when they are unsure about something. One GP explicitly stated that it is not necessary to know everything, that it is much more important to be able to make an initial assessment and ask for help when you reach your limits. The doctors trusted the APNs and felt that they would know when to ask for help.

*“But she knows her limits… And I have no doubt that she would ask. So far, I’ve never had the feeling that she crossed a line.”* (GP from practice B)

### GP’s lack of knowledge regarding the APN role

APNs and GPs agreed that the role is not yet well-known nor completely defined. The APNs stressed that most doctors do not know anything about the role of an APN, especially in primary care settings. However, they also admitted that sometimes they do not know themselves how exactly what their role looks like and that its development is an ongoing process.

*“But they did not even know who we are. They did not know that we have a master’s degree. […] In our country doctors have no idea what advanced practice nurses do.”* (APN, MScN)

*“And the role that I have today… I had to shape it myself. I did not come into this field of work and everything was prepared. I actually have to renegotiate it every day… with GPs, patients and other health professionals.”* (APN, further training completed)

The doctors agreed that they did not really know what to expect at the beginning of the collaboration. They were not familiar with the concept of advanced nursing practices beforehand. Hence, at the beginning, they did not know what APNs can do and what they are allowed to do.

*“Expectations were relatively vague. I did not know exactly what I was getting into… Now I see a little bit more what the possibilities and difficulties are.”* (GP from practice A)

### Political and legal obstacles in introducing the APN role

The nurses wished for more political and legal regulations, especially in regard of billing options and reimbursement. They would prefer an independent APN tariff, or alternatively, official legitimization of their use of the TARMED, together with the nursing tariff for home care under their own name. They understood why family practices are hesitant to hire them as long as there is no clear regulation provided for billing for their services.

*“However, it is difficult because the practices don’t know how to bill and whether we are profitable or not.”* (APN, in further training)

One APN referred to approaches of other countries, e.g. Israel, in which the government used a rigorous “top-down approach” to implement the role of nurse practitioners. Overall, most APNs desired more governance.

*“A political and legal framework and clarification is needed. There are many different projects in Switzerland, but everyone does it a little bit differently and it probably needs governance. So that we don't always have the discussion: What can I do and what am I allowed to do?”* (APN, MScN)

One APN explicitly stated that it is unpleasant to work under these conditions:

*“It's a stupid feeling, I know I'm doing something good, I know it makes sense, it also fits into current politics and economics, but I know I'm actually still kind of illegal. This grey area is annoying, it's unpleasant.”* (APN, further training completed)

The APNs agreed that a master’s degree as an entry level credential for advanced nursing practices makes sense, but stressed that further education to improve clinical skills, especially in the setting of primary care, with a lot of direct patient encounters, is helpful. Further, one APN showed fear regarding the future development of the APN role in Swiss primary care:

*“It scares me from a professional and political perspective, if APNs are only used as gap fillers in light of the lack of GPs. And then, if there are enough GPs again, one may dream, then we can leave… This really cannot be the solution! […] I just think that APNs have a right to exist in today’s demographic developments… an independent right to exist.”* (APN, further training completed)

The GPs expressed understanding of the difficult billing situation:

*“It doesn’t attract many people when you have to work in this grey area… it’s a bit annoying and the recognition of what you do is lacking if you cannot bill for your services yourself.”* (GP from practice B)

One GP also stressed the problem of the accountability and referred to the political situation:

*“If the APN does something, she has to report it to me, because at the end I am responsible. She can do it independently if it is her own responsibility… but that’s a political decision, whether this is desirable or not. In the current system we [physicians] are still responsible.”* (GP from practice A)

## Discussion

### Summary of the results

The APNs’ and GPs’ views resulted in five main themes: They considered themselves pioneers, developing a new model in primary care in order to shape and improve future health care ((1) pioneering spirit). Both nurses and doctors agreed on the additional value from the APN role, which was mainly seen in having more time for and a different approach to patients and bringing quality and flexibility to the practice ((2) added value from the APN role). They also emphasized the importance of asking for help when unsure about next steps ((3) awareness of limited knowledge and skills). The major barriers they cited are that Swiss doctors have little knowledge about nurses in advanced roles ((4) GP’s lack of knowledge regarding the APN role), and that further regulations would be important in order to foster role clarity and accountability ((5) political and legal obstacles in introducing the APN role).

### Interpretation and underlying mechanisms

Applying the PEPPA Plus framework, it can be seen that the APN role in Swiss primary care is, for the most part, still in the introduction stage. It appears to be essential to promote role clarity among stakeholders, especially among physicians. Even pioneering GPs who decided to engage an APN in their practice neither knew the role of an APN nor what to expect at the beginning of the collaboration. As the collaboration advanced, the additional benefits became more apparent and it seems that the APN role meets the needs of Swiss primary care with elderly, multimorbid and complex patients who require a holistic approach, more time, and often wish to be treated at home. However, to realize and see the benefits of the APN role, an initial extra effort is needed from both nurses and doctors. First, the GPs need to become familiar with the APN’s role and competencies. Then, both GPs and APNs need to build up mutual trust for an effective collaboration. Introducing the APN role in primary care is also characterised by pioneers with different ideas and approaches (“top-down” vs. “self-initiative”). This pioneering allows for flexibility in testing different models and helps to gather experiences and evidence about the advantages and/or disadvantages of each model in practice (“proof of concept”). It also provides a sound basis for future regulation in terms of scope of practice and reimbursement schemes.

Certain aspects of the implementation stage, such as educational resources, have been put in place. Several competing master’s programs, as well as further training options, are available in Switzerland, some with a specific focus on primary care. These programs are constantly evolving and aim to include more clinical skills and practical experiences during the studies. However, political and legal resources such as well-defined and specific laws are still lacking; reimbursement, curricula, accountability and scope of practice are not yet clearly regulated.

**Fig. 1:**
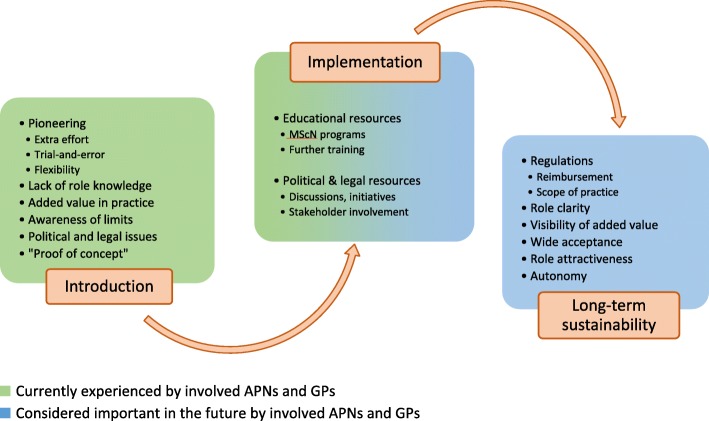
Elements during APN role development in Swiss primary care (adapted from PEPPA Plus)

Our study results reflect the ongoing, controversial political debates about the nurses’ demands for more professional autonomy and regulations. According to our participants, lack of governance might hinder further implementation of APNs in practice. However, as mentioned in the background, initial political steps towards more autonomy for nurses were rejected by the national council and the ongoing popular initiative (“Pflegeinitiative”) is highly debated among politicians and other stakeholders. For instance, the initiative is not supported by the federal council because an increase in volume and costs is feared if uncoordinated and overhasty steps are taken [29]. Yet, the federal council recognises the importance and value of nurses as an indispensable part of ambulatory care. They are willing to examine and develop further measures, but point out that there is already a legal basis to strengthen the nurses’ role in primary care [29, 30]. Indeed, law articles promoting and enabling pilot projects to try out new models of care with the goal to reduce costs exist and further articles will be implemented [30, 31]. Moreover, health insurance companies show interest in innovative pioneer projects and flexibility about pragmatic billing solutions. In the future, further regulation will be necessary, but exaggerated and premature political and legal demands might hamper and set back the implementation of advanced nursing practices in Swiss primary care. Our results and interpretations are summarised and illustrated in Fig. 1.

### Comparison to other studies

Results from Steinbrüchel et al. [24] showed that uninvolved GPs know little about the APN role but consider the financial aspect as crucial. They also highlighted that the quality of the APNs’ work was key in order to implement the role. Our study gives a more detailed view from doctors who actually work with APNs. At the beginning of the collaboration, they were also not familiar with the role, but they did see the value of APNs, and observed an improvement of the quality of care in their practice over time. However, the financing situation remains ambivalent and incoherent.

In other European countries, similar studies had been done when the APN role was introduced in primary care. Wilson et al. [32] explored the views of GPs on developing the APN role in general practices in Great Britain. In their work, the financial structures of general practices were also mentioned as one of the major constraints on implementing the APN role. Furthermore, the GPs expressed fear of a more complex caseload because the nurses might take over all the *easy* consultations. This fear was shared by some Dutch GPs interviewed by van der Biezen et al. [33], but participants in our study did not share this view. In addition, van der Biezen et al. showed that improvement in quality of care and the ability to offer additional services (i.e. technical care) were important factors driving GPs to employ APNs. In another study from the Netherlands, Lovink et al. [34] found additional value from the APN role in a patient-centred view and in improved continuity of care. These aspects were also emphasized by our interviewees. When the APN role was introduced in Swedish primary care, Lindblad et al. [35] interviewed APNs and GPs. They stressed the importance of mutual trust: the APN should be able to ask for help when needed, in exchange the GPs needed to be sure that s/he would do so. Furthermore, the GPs and nurses considered it the APN’s role to serve an extra resource to increase the availability of care for patients. These results are in accordance with our own findings. The importance of prior knowledge of the role during the implementation process was highlighted by our participants and is illustrated by the previous work of Sangster-Gormley et al. [36]: They identified prior knowledge as one of the main drivers for acceptance and involvement of the APN role in primary care. A review by Schadewaldt et al. [37] confirmed this finding; GPs with previous experience of collaborating with an APN have a more positive attitude towards the role. In our study, the APNs demanded more regulation in order to increase the attractiveness of their profession and to facilitate further implementation of their role. Maier et al. [38] found that regulations may enable introduction of advanced nursing practices, but only if they are up-to-date and not restrictive. Barnes et al. [39] showed that removing restrictions on APNs’ scope of practice and offering higher reimbursement rates might indeed increase the share of APNs working in primary care settings. However, this study was conducted in the US and its transferability to the Swiss context may be limited.

### Strengths & Limitations

We conducted individual interviews with only four doctors and two APNs. We did not conduct focus groups with GPs, as they were difficult to recruit outside the ongoing evaluations due to busy work schedules and other projects. Nonetheless, in the APN focus groups we reached a comprehensive sample of almost all APNs currently working in Swiss primary care. The interviews allowed for an in-depth discussion with individuals, while the focus groups facilitated an exchange between people from various settings and with different experiences. Regardless, the generalisability and transferability of our results might be limited due the small sample size. Furthermore, our study design is prone to selection bias in the sense that only people already involved in projects and motivated to participate were part of the study (though this is not unusual for a pioneer setting). Our study is also prone to self-reporting bias since the participants were interviewed about their own activities and achievements. This might compromise the credibility of the results. To reduce researcher bias and ensure trustworthy, the data analysis followed a structured and rigorous approach using a theoretical framework.

### Implications & Outlook

Promoting role clarity and understanding could facilitate the introduction of the APN role in Swiss primary care. Pioneering and small-scale projects are important to demonstrate the feasibility of this new model and to gather evidence of the added value from and the potential scope of practice of the APN role in ambulatory care. Pilot projects show how nurses in advanced roles can help tackle the impending challenges in primary care and may contribute to acknowledgment of the role by a wider audience including physicians, patients, politicians and insurers. Further regulations in terms of clear scope of practice, accountability and reimbursement are important but need to be planned carefully and implemented together with all relevant stakeholders, i.e., politicians, educators, health professionals and insurers. Premature and extensive demands before the feasibility and value of the concept are demonstrated could jeopardize the long-term sustainability of the APN role in Swiss primary care. Our study might provide valuable insights for other countries with similar health care systems such as Germany or Austria, which are also in the early stages of introducing the APN role to primary care.

Further studies are needed to validate and quantify the additional benefits, scope of practice and costs of the APN role in the Swiss health care system. In addition, the views of relevant stakeholders (e.g. patients and politicians) on steps for further implementation of such a role in the system must be studied. Lastly, the regulations most suitable to Switzerland and the times at which they can realistically be implemented must be determined.

## Conclusions

The early phase of introducing APNs to Swiss primary care is characterised by heterogeneous, small-scale projects of pioneering GPs and APNs recognising the added value and limits of APNs despite a lack of governance and knowledge regarding the APN role among GPs. Experiences gained from ongoing projects provide elements of good practice for political discussions and regulations.

## Supplementary information

**Additional file 1.** Interview guides.

**Additional file 2.** Codebook.

## Data Availability

Due to the sensitivity of the data (e.g. practice billing mechanisms), the datasets are not publicly available. Parts of the transcripts are available from the authors upon reasonable request and with permission of the study participants.

## Abbreviations

ACP: Advance Care Planning
APN: Advanced Practice Nurse
CNS: Clinical Nurse Specialist
GLN: Global Location Number
GP: General Practitioner
ICN: International Council of Nurses
NP: Nurse Practitioner
TARMED: tarif médical (medical tariff)

## References

[1] Nolte E, Knai C. Assessing chronic disease management in European health systems. Country Reports. 2015;37(1):1–7. 29035490

[2] Maier CB, Aiken LH, Busse R. Nurses in advanced roles in primary care: policy levers to implementation. OECD Health Working Paper Paris. 2017;98:13–43.

[3] Delamaire M-L, Lafortune G. Nurses in advanced roles: description and evaluation of experiences in 12 developed countries. OECD Health Working Papers. 2010;54:29–31.

[4] Puertas EB, Arósquipa C, Gutiérrez D (2013). Factors that influence a career choice in primary care among medical students from high-, middle-, and low-income countries: a systematic review. Rev Panam Salud Publ.

[5] Naylor MD, Kurtzman ET (2010). The role of nurse practitioners in reinventing primary care. Health Affair.

[6] Sheer B, Wong FKY (2008). The development of advanced nursing practice globally. J Nurs Scholarship..

[7] International Council of Nurses. Nurse Practitioner / Advanced Practice Network: Definitions and Characteristics of the Role. https://international.aanp.org/Practice/APNRoles. Accessed 12 Dec 2018.

[8] Bryant-Lukosius D, Spichiger E, Martin J, Stoll H, Kellerhals SD, Fliedner M (2016). Framework for evaluating the impact of advanced practice nursing roles. J Nurs Scholarship.

[9] Martínez-González NA, Djalali S, Tandjung R, Huber-Geismann F, Markun S, Wensing M (2014). Substitution of physicians by nurses in primary care: a systematic review and meta-analysis. BMC Health Serv Res.

[10] Laurant M, van der Biezen M, Wijers N, Watananirun K, Kontopantelis E, van Vught AJAH. Nurses as substitutes for doctors in primary care. Cochrane Database Syst Rev. 2018;(7):CD001271. 10.1002/14651858.CD001271.pub3. 10.1002/14651858.CD001271.pub3 PMC6367893 30011347

[11] Jakimowicz M, Williams D, Stankiewicz G (2017). A systematic review of experiences of advanced practice nursing in general practice. BMC Nurs.

[12] Schober M (2016). Introduction to advanced nursing practice: springer.

[13] Sottas B, Brügger S (2012). Ansprechstrukturen: Perspektivenwechsel und Grenzverschiebungen in der Grundversorgung: Careum.

[14] Maier CB, Aiken LH (2016). Task shifting from physicians to nurses in primary care in 39 countries: a cross-country comparative study. Eur J Pub Health.

[15] De Pietro C, Camenzind P, Sturny I, Crivelli L, Edwards-Garavoglia S, Spranger A, et al. Health Systems in Transition. Health. 2015;17(4):104–6. 26766626

[16] Spitex Schweiz: Kassenpflichtige Leistungen. https://www.spitex.ch/Nonprofit-Spitex/Tarife/Kassenpflichtige-Leistungen/PaAVw/. Accessed 9 Jan 2019.

[17] De Geest S, Moons P, Callens B, Gut C, Lindpaintner L, Spirig R (2008). Introducing advanced practice nurses/nurse practitioners in health care systems: a framework for reflection and analysis. Swiss Med Wkly.

[18] Morin D, Ramelet A-S, Shaha M (2013). Vision suisse romande de la pratique infirmière avancée. Rech Soins Infirm.

[19] swissANP: Positionspaper ANP CH. http://www.swiss-anp.ch/fileadmin/3_ANP_Berufsrolle/2012_EckpunktepapierANP.pdf. Accessed 16 Dec 2018.

[20] Kieser U (2016). Advanced practice nurse und clinical nurse specialist–neue Entwicklungen bei Pflegefachpersonen. Pflegerecht..

[21] Das Schweizer Parlament. Parlamentarische Initiative Joder Rudolf: Gesetzliche Anerkennung der Verantwortung der Pflege. https://www.parlament.ch/de/ratsbetrieb/amtliches-bulletin/amtliches-bulletin-die-verhandlungen?SubjectId=37189. Accessed 9 Jan 2019.

[22] Pflegeinitiative: Volksinitiative für eine starke Pflege. http://www.pflegeinitiative.ch. Accessed 9 Jan 2019.

[23] Kambli K, Flach D, Schwendimann R, Cignacco E (2015). Health care provision in a Swiss urban walk-in-clinic. Is advanced nursing practice a solution for a new model in primary care?/Gesundheitsversorgung in einer städtischen walk-in-praxis in der Schweiz. Ist advanced nursing practice ein neues Modell in der Grundversorgung?. Int J Health Prof.

[24] Steinbrüchel-Boesch C, Rosemann T, Spirig R. Neue Zusammenarbeitsformen mit Advanced Practice Nurses in der Grundversorgung aus Sicht von Hausärzten–eine qualitativ-explorative Studie. Praxis. 2017. 10.1024/1661-8157/a002658 28443716

[25] Laurant M, Reeves D, Hermens R, Braspenning J, Grol R, Sibbald B. Substitution of doctors by nurses in primary care. Cochrane Database Syst Rev. 2005;(2):CD001271. 10.1002/14651858.CD001271.pub2. 10.1002/14651858.CD001271.pub2 15846614

[26] Fereday J, Muir-Cochrane E (2006). Demonstrating rigor using thematic analysis: a hybrid approach of inductive and deductive coding and theme development. Int J Qual Meth.

[27] Crabtree BF, Miller WL (1999). Doing qualitative research: sage publications.

[28] Boyatzis RE (1998). Transforming qualitative information: thematic analysis and code development: sage.

[29] Der Bundesrat. Das Portal der Schweizer Regierung: Bundesrat lehnt «Pflegeinitiative» ab. https://www.admin.ch/gov/de/start/dokumentation/medienmitteilungen/bundesrat.msg-id-70065.html. Accessed 9 Jan 2019.

[30] Der Bundesrat. Das Portal der Schweizer Regierung: Kostendämpfung im Gesundheitswesen: Bundesrat nimmt alle Akteure in die Pflicht. https://www.admin.ch/gov/de/start/dokumentation/medienmitteilungen.msg-id-72182.html. Accessed 9 Jan 2019.

[31] Der Bundesrat. Das Portal der Schweizer Regierung: Bundesverfassung der Schweizerischen Eidgenossenschaft: Art. 117a Medizinische Grundversorgung. https://www.admin.ch/opc/de/classified-compilation/19995395/index.html#a117a. Accessed 9 Jan 2019.

[32] Wilson A, Pearson D, Hassey A (2002). Barriers to developing the nurse practitioner role in primary care—the GP perspective. Fam Pract.

[33] van der Biezen M, Derckx E, Wensing M, Laurant M (2017). Factors influencing decision of general practitioners and managers to train and employ a nurse practitioner or physician assistant in primary care: a qualitative study. BMC Fam Pract.

[34] Lovink MH, van Vught AJAH, Persoon A, Schoonhoven L, Koopmans RTCM, Laurant MGH (2018). Skill mix change between general practitioners, nurse practitioners, physician assistants and nurses in primary healthcare for older people: a qualitative study. BMC Fam Pract.

[35] Lindblad E, Hallman EB, Gillsjö C, Lindblad U, Fagerström L (2010). Experiences of the new role of advanced practice nurses in Swedish primary health care—a qualitative study. Int J Nurs Pract.

[36] Sangster-Gormley E, Martin-Misener R, Burge F (2013). A case study of nurse practitioner role implementation in primary care: what happens when new roles are introduced?. BMC Nurs.

[37] Schadewaldt V, McInnes E, Hiller JE, Gardner A (2013). Views and experiences of nurse practitioners and medical practitioners with collaborative practice in primary health care – an integrative review. BMC Fam Pract.

[38] Maier CB (2015). The role of governance in implementing task-shifting from physicians to nurses in advanced roles in Europe, US, Canada, New Zealand and Australia. Health Policy.

[39] Barnes H, Maier CB, Altares Sarik D, Germack HD, Aiken LH, McHugh MD (2017). Effects of regulation and payment policies on nurse practitioners’ clinical practices. Med Care Res Rev.

