# Supplementary material for: Advanced practice nurses’ and general practitioners’ first experiences with introducing the advanced practice nurse role to Swiss primary care: a qualitative study
Source: BMC Fam Pract. 2019 Nov 27;20:163. doi: 10.1186/s12875-019-1055-z (PMC6880366; doi:10.1186/s12875-019-1055-z)
Supplement: Supplementary file 1 — Additional file 1. Interview guides. [file 12875_2019_1055_MOESM1_ESM.docx]

**Additional file 1: Interview guides**

Interview guide for the GPs of practice A

*At the start of the project in August 2017*

1. In general, what are your expectations regarding this project?
2. How do you see future task sharing between the APN and yourself?
   1. Which tasks / patients are particularly suitable to be taken over by the APN?
   2. Which tasks / patients belong in the hands of a doctor? What are the limits for an APN?
   3. Do you expect that the task sharing will change over time?
3. How do you see the future organisation and collaboration within the practice working?
   1. How will the decision of who does which tasks and who sees which patients be made?
   2. How do you expect the collaboration to work?
   3. How will you mentor the APN?
4. Which competencies are needed for an APN to work in primary care?
   1. Does the APN have these competencies?
   2. Are there any gaps regarding her knowledge and skills?
   3. What do you want to teach her?
5. How do you expect the patients and the population to react to / accept the APN?
6. What are possible challenges and difficulties for an APN in primary care, and for this project specifically?
7. What are your expectations in terms of the benefits…
   1. for yourself and the practice?
   2. for patients and their relatives?
   3. for the canton and the health care system as a whole?
   4. for others?
8. What is necessary for this project to be successful?

*About six months after the project started*

1. In general, what were your expectations regarding this project, and how have they changed?
2. How do you assess the task sharing between the APN and yourself?
   1. Which tasks / patients are particularly suitable to be taken over by the APN?
   2. Which tasks / patients belong in the hands of a doctor? Where are the limits for an APN?
   3. How did the task-sharing change over time?
3. How does the organisation and collaboration within the practice work?
   1. How are the decisions of who does which tasks and who sees which patients made?
   2. How is the collaboration between you and the APN going?
   3. How is the mentoring working / progressing?
4. Which competencies are needed for an APN to work in primary care?
   1. Does the APN have these competencies?
   2. Are there any gaps regarding her knowledge and skills?
   3. What do you still want to teach her?
5. How are your patients and the population reacting to / accepting the APN?
6. What are challenges and difficulties for an APN in primary care and for this project specifically?
7. What are the benefits of this project…
   1. for yourself and the practice?
   2. for patients and their relatives?
   3. for the canton and the health care system as a whole?
   4. for others?
8. What is necessary for this project to continue successfully?

Interview guides for the APN of practice A

*At the start of the project in August 2017*

1. In general, what are your expectations regarding this project?
2. How do you see the future task sharing between the GPs and yourself?
   1. Which tasks / patients are particularly suitable to be taken over by you?
   2. Which tasks / patients belong in the hands of a doctor? Where are your limits?
   3. Do you expect that the task sharing will change over time?
3. How do you see the future organisation and collaboration within the practice working?
   1. How will the decision of who does which tasks and who sees which patients be made?
   2. What are your expectations regarding the collaboration with other team members?
   3. How should mentoring be structured and performed?
   4. What is necessary for an effective collaboration?
4. Which competencies are needed for an APN to work in primary care?
   1. Do you have these competencies?
   2. Are there any gaps in your knowledge and skills?
   3. What are your expectations in terms of mentoring?
5. How do you expect the acceptance of your role among doctors, patients and the population to be?
6. What are possible challenges and difficulties for an APN in primary care and for this project specifically?
7. What are your expectations in terms of the benefits…
   1. for yourself and the practice?
   2. for patients and their relatives?
   3. for the canton and the health care system as a whole?
   4. for others?
8. What is necessary for this project to be successful?

*About six months after the project started*

1. In general, what were your expectations regarding this project, and what has changed?
2. How do you assess the task sharing between the GPs and yourself?
   1. Which tasks / patients are particularly suitable to be taken over by you?
   2. Which tasks / patients belong in the hands of a doctor? Where are your limits?
   3. Has the task sharing changed over time?
3. How do you assess the organisation and collaboration within the practice?
   1. How is the decision of who does which tasks and who sees which patients made?
   2. How is the collaboration going?
   3. How is the mentoring structured and performed?
   4. What is necessary for an effective collaboration?
4. Which competencies are needed for an APN to work in primary care?
   1. Do you have these competencies?
   2. Are there any gaps in your knowledge and skills?
   3. What were your expectations in terms of mentoring, and how is it going?
5. How is the acceptance among doctors, patients and the population?
6. What are challenges and difficulties for an APN in primary care, and for this project specifically?
7. What are the benefits of this project…
   1. for yourself? for the practice?
   2. for the patients and their relatives?
   3. for the canton and the health care system as a whole?
   4. for others?
8. What is necessary for this project to continue successfully?

Interview guide for the GPs of practice B

*April 2018, about two years after the project started*

1. What was your motivation to engage an APN? What were the expectations?
2. How is the task sharing between you and the APN? How has this evolved over time?
   1. Which tasks / patients are particularly suitable for the APN?
   2. Where are the limits? Which tasks / patients definitely belong in the hands of a doctor?
3. Which competencies does an APN need to work in primary care?
   1. What were the gaps at the beginning? What did she have to learn?
   2. The APN works according to a “nursing” concept. To what extent is this suitable in a primary care practice, and were any adjustments necessary?
   3. How important was the mentorship and how was it done?
   4. How would you describe the APN’s development of competencies in terms of "entrustable professional activities"?
4. How do you assess the task sharing and organisation within (and outside) the practice?
   1. Is there still room for improvement?
   2. Are there specific difficulties faced by APNs in a primary care practice?
5. What is your impression of the level of acceptance among health professionals, patients, institutions and in the population?
6. What were the three greatest successes? What were the three biggest challenges and how were they resolved?
7. What do you consider the benefits to be for…
   1. the APN herself?
   2. the practice?
   3. for other GPs? For other institutions and home care organisations?
   4. the patients and relatives? For the region?
   5. for the APN community in Switzerland? For politics (local government, health policy)?

Interview guide for the APN of practice B

*March 2018, about two years after the project started*

1. What were the conditions and your expectations when starting this project? Were your expectations fulfilled? At what point did the “pilot” character disappear?
2. Which patients / tasks are particularly suitable for an APN?
3. How does task sharing and organization work in the practice?
   1. How does the triage work?
   2. Has this changed over time?
4. Where do you see your limits? Which patients / tasks belong in the hands of a doctor?
5. When do you need the help / support of a doctor? How is / was the mentoring performed?
   1. What is particularly important?
   2. What has changed over time?
6. How was collaboration with the GPs, other health professionals and institutions established, and how has it changed over time?
   1. What were facilitators and barriers?
7. What competencies does an APN need to work in primary care?
   1. What were the gaps at the beginning?
   2. What can be taken from the “nursing concept” for effective practice?
8. What were three successes or factors for success? What were three difficulties and how were they resolved?
9. What is your impression of the level of acceptance among patients, health professionals and in the population? And why?
10. How do you assess the benefits of your role for…
    1. yourself?
    2. the practice?
    3. the GPs? For other institutions and home-care organisations?
    4. patients and their relatives? For the region?
    5. for the APN community in Switzerland? For politics (local government, health policy)?

Interview guide focus group discussions

1. Can you please introduce yourself?
   1. Name, current work position (since when)
   2. How did you get this position? How did this collaboration start? What is your role?
   3. What is your (educational) background?
   4. What is/was your motivation to become an APN (and to work in primary care)?
2. Education:
   1. Advantages and disadvantages of the different educational programs in Switzerland?
   2. How is the education in comparison to other countries?
   3. How do you refer to yourself? (after completing your education)
3. Tasks and task sharing in a primary care practice
   1. What are the tasks of an APN? Which tasks are suitable for an APN? What competencies are needed?
   2. Where are the limits? When do you need a doctor?
   3. Interprofessional collaboration: Who does what? Are there any overlaps or is it complementary? Does it change over time?
   4. How are tasks allocated?
4. Supervision / Mentoring
   1. Is there any supervision by the GP? If so, how does it work?
   2. Which tasks can you perform independently?
   3. Do you make diagnoses? Do you order tests? Do you prescribe and / or write referrals?
5. Acceptance
   1. Within the practice team
   2. Among patients, relatives and the population
   3. Among other health professionals and at institutions
6. Legal aspects / billing options
   1. Prescription, referrals
   2. How do you bill for your services?
   3. What do you hope for in the future?
   4. Responsibility, accountability?
7. Final statements: Who are you and what are you doing as an APN? What are your future ideas / plans?
