# Supplementary material for: Advanced practice nurses’ and general practitioners’ first experiences with introducing the advanced practice nurse role to Swiss primary care: a qualitative study
Source: BMC Fam Pract. 2019 Nov 27;20:163. doi: 10.1186/s12875-019-1055-z (PMC6880366; doi:10.1186/s12875-019-1055-z)
Supplement: Supplementary file 2 — Additional file 2. Codebook. [file 12875_2019_1055_MOESM2_ESM.docx]

**Additional file 2: Codebook**

*Based on the PEPPA Plus Framework by Bryant-Lukosius et al. (2016)*

| *Code category 1* |  |
| --- | --- |
| Label | Competencies |
| Definition | Core competencies of ANP by Hamric |
| Codes (including description and examples) | Competencies:   - Clinical practice - Ethical decision making - Guidance & coaching - Consultation - Evidence-based practice - Leadership - Collaboration - Research |

| *Code category 2* |  |
| --- | --- |
| Label | Role |
| Definition | Type of APN role |
| Codes (including description and examples) | Determine unique ANP role characteristics (🡪 APN in Swiss primary care)  Role clarity  Clinical Nurse Specialist & Nurse Practitioner:   - CNS: in-depth expertise and experience in a specialized area of practice (e.g. oncology) - NP: expanded scope of practice that permits diagnosing, prescribing, treating, referring, admitting / discharging patients (from hospitals) |

| *Code category 3* |  |
| --- | --- |
| Label | Stage |
| Definition | Stages of APN role development |
| Codes (including description and examples) | Introduction:   - Identifying the patient population - Engaging stakeholders in role design process - Establishing the need for the ANP role - Role goals - Defining ANP role and competencies - Planning the implementation   Implementation:   - Introduction of the role - Putting into place resources necessary to support ANP role development (funding, education, legislation etc.) - Recruitment & hiring - Assess barriers to role implementation - Impact   Long-term sustainability:   - Ongoing monitoring and evaluation to determine the extent of role integration - New and continuing need for the role & impact |

| *Code category 4* |  |
| --- | --- |
| Label | Structures |
| Definition | Factors that influence how APN roles are implemented |
| Codes (including description and examples) | Practical support: continuing education, mentorship, peer support and communication support  Human resources:   - Personal & professional characteristics of the APN: education, experience, skills, knowledge, competence, confidence - ANP role competencies & educational programs - Patient characteristics (age, conditions, etc.) - Supply & demand of physicians, other healthcare providers and APNs - Patient, team, stakeholders: awareness, understanding, acceptance and support of the APN role   Physical resources:   - Office space - Patient referral area   Characteristics of the practice environment:   - Organizational: size, structure (horizontal vs. vertical), reporting mechanisms - Cultural: expectations of APN role, organizational culture and values (e.g. interprofessional care, patient-centred) - Political: legislation, policies - Economic: funding, reimbursement |

| *Code category 5* |  |
| --- | --- |
| Label | Processes |
| Definition | APN role implementation or types of APN services and interventions provided and how they are delivered |
| Codes (including description and examples) | Activities related to ANP role competencies by Hamric et al.  “Dose effect”: amount of APN interventions   - Frequency / intensity of APN – patient interactions - Responsiveness of the target population to the intervention - Factors that influence the “dose”, e.g. APN’s education & experience |

| *Code category 6* |  |
| --- | --- |
| Label | Outcomes |
| Definition | The accumulated effects or results of APN services and interventions |
| Codes (including description and examples) | Perspectives:   - Patients & families - Communities & populations - APN - Healthcare providers / team - Organization - The broader healthcare system   Categories:   - Patient & family: health status (quality of life), health behaviours (self-management), perception (satisfaction) - Quality of care: patient safety, processes of care (medication use, continuity of care), access to care - Health care provider & stakeholder: acceptance and satisfaction, job satisfaction - Organization: recruitment and retention of APN - Healthcare use & costs (savings)   Short-term outcomes:   - Awareness, understanding and acceptance of the APN role   Intermediate outcomes: (within 3 years)   - Improvement in patient health behaviours and/or team function   Long-term outcomes: (after 4 to 5 years)   - Sustained improvements in patient and healthcare system outcomes - APN role integration |
